# The Influence of Body Position on Cerebrospinal Fluid Pressure Gradient and Movement in Cats with Normal and Impaired Craniospinal Communication

**DOI:** 10.1371/journal.pone.0095229

**Published:** 2014-04-18

**Authors:** Marijan Klarica, Milan Radoš, Gorislav Erceg, Antonio Petošić, Ivana Jurjević, Darko Orešković

**Affiliations:** 1 Department of Pharmacology and Croatian Institute for Brain Research, School of Medicine University of Zagreb, Zagreb, Croatia; 2 Department of Electroacoustics, Faculty of Electrical Engineering and Computing University of Zagreb, Zagreb, Croatia; 3 Department of Molecular Biology, Ruđer Bošković Institute, Zagreb, Croatia; University of Maryland, College Park, United States of America

## Abstract

Intracranial hypertension is a severe therapeutic problem, as there is insufficient knowledge about the physiology of cerebrospinal fluid (CSF) pressure. In this paper a new CSF pressure regulation hypothesis is proposed. According to this hypothesis, the CSF pressure depends on the laws of fluid mechanics and on the anatomical characteristics inside the cranial and spinal space, and not, as is today generally believed, on CSF secretion, circulation and absorption. The volume and pressure changes in the newly developed CSF model, which by its anatomical dimensions and basic biophysical features imitates the craniospinal system in cats, are compared to those obtained on cats with and without the blockade of craniospinal communication in different body positions. During verticalization, a long-lasting occurrence of negative CSF pressure inside the cranium in animals with normal cranio-spinal communication was observed. CSF pressure gradients change depending on the body position, but those gradients do not enable unidirectional CSF circulation from the hypothetical site of secretion to the site of absorption in any of them. Thus, our results indicate the existence of new physiological/pathophysiological correlations between intracranial fluids, which opens up the possibility of new therapeutic approaches to intracranial hypertension.

## Introduction

According to the generally accepted classical hypothesis of cerebrospinal fluid (CSF) physiology, CSF is actively formed mostly inside the ventricles, than it flows unidirectionally through the system of ventricles to the cisterna magna (CM) [1]–[3]. From there, one undetermined part of the volume flows around the spinal medulla, while the rest of the CSF circulates from the infratentorial subarachnoid space through the tentorial apperture into the supratentorial subarachnoid space. At the end, the largest part of the CSF is passively absorbed through the arachnoid villae into the dural venous sinuses of the brain convexity. Except this main site of absorption, there is a large amount of literature which suggests that the absorption of CSF occurs from the subarachnoid space to the lymphatic system [4]–[7]. Thus, CSF physiology is based on three key premises: the active CSF formation (secretion), the passive CSF absorption, and the unidirectional CSF flow from the place of formation to the place of absorption. However, for the mentioned theory of CSF physiology to be true, the key is also the fourth premise: the existence of a hydrostatic pressure gradient inside the CSF system, without which there can be neither CSF circulation nor passive CSF absorption.

It is clear that the pressure gradient which enables the unidirectional CSF circulation should include the following: the highest pressure value has to be at the site of the CSF secretion inside the ventricles, it should be somewhat lower in the cisterna magna and around the subarachnoid space, and the lowest at the site where CSF is passively absorbed. Furthermore, passive CSF absorption is inconceivable if the CSF pressure is not higher than the pressure inside the venous sinuses. The common opinion is that CSF absorption becomes operative only if the CSF pressure is above 50–70 mm cm H_2_O, which is the pressure that produced the hydrostatic gradient pressure between the CSF space and the venous blood in the dural sinuses [2]. While the pressure gradient related to CSF absorption was already investigated [2], [8], the gradients between the pressures that are crucial for sustaining CSF circulation inside the CSF space were not scientifically explored or seriously analyzed.

CSF pressure is usually measured while a person is lying in a horizontal recumbent position. Normal CSF pressure values, in that case, are around 15 cm H_2_O, and the pressure is the same along the spinal subarachnoid space and inside the cranium [1]. Thus, according to the data that is available, in a horizontal position there is no hydrostatic pressure gradient that would be necessary in order for CSF to circulate inside the cranial and spinal CSF space.

In sitting subjects (upright position), Masserman [9], Loman [10], Loman et al. [11], Von Storch et al. [12], O'Connell [13], Magnaes [14], [15] and many others all found fluid pressure to be at the atmospheric pressure level in the upper cervical region or at the level of the foramen magnum. Inside the lumbar region, the pressure is positive and its value corresponds to the distance from the CM to the measuring site in the lumbar region. This fact raises a question: How can CSF circulate through the spinal subarachnoid space from the CM to the lumbar region while we are in an upright position? That circulation direction would be contrary to the hydrostatic pressure gradient.

Furthermore, it is known that the change in body position (from horizontal to upright, head up or sitting position) is followed by a transient fall in intracranial pressure (ICP) [14], [15] to the subatmospheric value, and it results in a new pressure gradient along the craniospinal axis. It is generally accepted that this decrease lasts for a very short period of time, and that the ICP remains positive. Namely, according to the classical hypothesis, in physiological conditions the rate of CSF secretion (V_f_) should be the same as the rate of absorption. Otherwise, when absorption is lesser than secretion, CSF will accumulate and increase the intracranial pressure [16]. The famous formula of professor Marmarou [16] links the classical concept of CSF physiology and intracranial pressure:
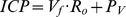
where R_o_ =  the resistance to the flow (circulation) of CSF along the CSF system; P_v_ =  the resistance to absorption of the CSF into the venous sinuses (blood circulation).

Marmarou's formula and the classical concept of CSF physiology suggest that the pressure has to be positive because the CSF secrets, flows unidirectionally, and absorbs all the time. According to the Monroe-Kellie doctrine, ICP depends on the interaction between three volumes: volume of the brain, volume of blood and volume of CSF. It is generally accepted that, when the head is above the heart level, intracranial venous blood is redistributed to the lower parts of the body, whereby venous vessels collapse [1], [17], [18] and intracranial CSF pressure decreases. It is also assumed that under such conditions a part of the intracranial CSF volume flows into the spinal CSF space [19]. Thus, the intracranial CSF pressure temporarily decreases and the intracranial compliance increases due to changes in intracranial blood and CSF volumes [13], [20], [21]. In other words, according to the Monroe-Kellies doctrine, the pressure inside the cranium could not change (increase or decrease) if a change in at least one of the three volumes filling the cranium did not occur.

However, the results obtained in our recent study indicate that a decrease in intracranial pressure in an upright position is possible without the displacement of cranial CSF volume to hydrostatically lower body parts [22]. It was possible to notice this because we have developed a new model of the CSF space in which we have imitated the well-known biophysical features of the cranial and spinal dura mater (see Materials and Methods; [22]). Intracranial dura is fixed to the bone surface, therefore, intradural volume inside the cranium is practically unchangeable. Contrary to that, in the spine, dura is only partially fixed to the bone and so spinal intradural volume can be significantly changed due to its distensibility [23]–[25]. The model of the “CSF system” consists of a distensible “spinal” part and a nondistensible “cranial” part filled with artificial CSF, which was constructed to imitate the anatomical dimensions and biophysical characteristics (distensible/nondistensible) of the CSF system in cats. It is necessary to stress that in this model there is no “CSF secretion, circulation and absorption”, and that the “CSF pressure” inside the model behaves entirely in accordance with fluid mechanics.

Based on the aforementioned unresolved questions, as well as the recently published doubts about the classical concept of CSF secretion, unidirectional circulation and absorption, and also the newly proposed concept of CSF physiology [26]–[28], in this article we wanted to critically examine the relation of the CSF pressure gradient and the classical hypothesis of CSF physiology. If CSF pressure behaves according to the law of fluid mechanics, and is not induced by CSF secretion, we expect that the negative (subatmospheric) CSF pressure will occur immediately after change of body position and will stay negative inside the cranium in an upright position the entire time of the experiment.

## Materials and Methods

### Animal experiments

The study was performed on male and female adult cats (2.2–3.4 kg body weight). The animals were kept in cages with natural light-dark cycles and had access to water and food (SP215 Feline, Hill's Pet Nutrition Inc., Topeka, KS, USA).

### Ethics statement

The animals were in quarantine for 30 days and the experiments were performed in accordance with the Croatian Animal Welfare Act. The protocol was approved by the Ethics Committee of the University of Zagreb Medical School (Approval No. 04-76/2006-18). Experiments shown in manuscript were performed more than 8 years ago. In that time in Croatia, Croatian Animal Welfare Act allowed us to obtain experimental animals from private owners (domestic breeding). However, today in Croatia we have a new Animal Welfare Act by which it is possible to obtain experimental animals only from official suppliers (and we are currently doing so). The owners were verbally informed about the experimental protocol which was previously approved by official Ethical committee (written consent form was not needed in that time). All efforts were made to minimize suffering, and all surgery according to protocol was performed under anesthesia.

The cats were anaesthetized with α-chloralose (Fluka; 100 mg/kg i.p.) and fixed in a stereotaxic head holder (David Kopf, Tununga, CA, USA) in the sphinx position. The femoral artery was cannulated, the blood pressure was recorded via a T-connector, and samples of blood were taken for analysis of the blood gases. No significant changes, either in blood pressure or blood gases, were observed during these experiments on cats, which continued breathing spontaneously under the chloralose anesthesia. A stainless steel cannula (0.9 mm ID) was introduced into the left lateral ventricle at 2 mm lateral and 15 mm anterior to the stereotaxic zero point, and 10–12 mm below the dural surface. A second cannula was placed in the right lateral ventricle (LV) (at same position as the cannula in the left LV; [29]). The cannula in the right LV was used for the measurement of intracranial CSF pressures. In order to measure the spinal CSF pressure in the lumbar region, a laminectomy (5×10 mm) of the L_3_ vertebra was performed. After incision of the spinal dura and arachnoidea, a third plastic cannula (0.9 mm ID) was introduced into the subarachnoid space. Leakage of CSF was prevented by applying cyanoacrylate glue to the dura around the cannula. Bone openings in the cranium and vertebra were hermetically closed by the application of a dental acrylate.

**Figure 1:**
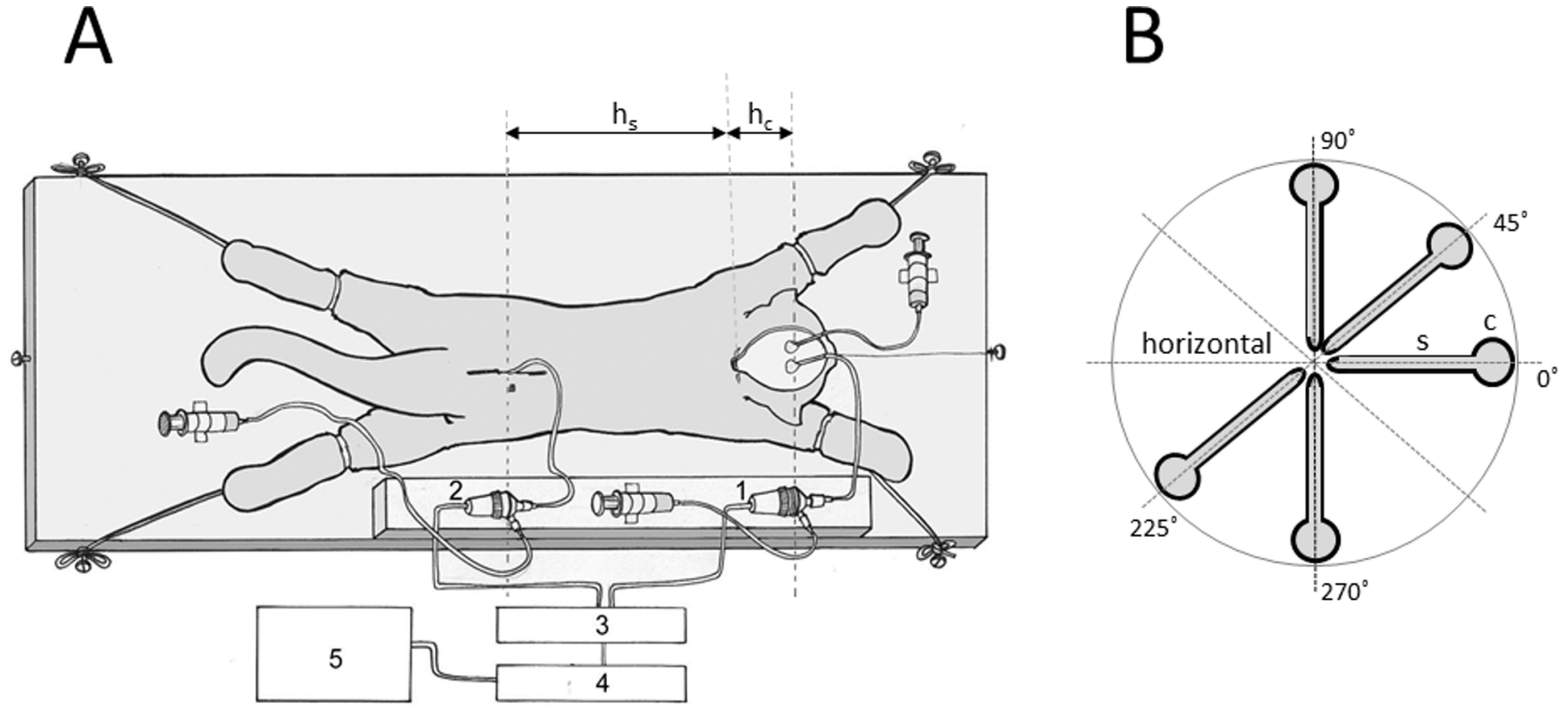
Scheme of a cat experimental model. A. 1 – pressure transducer connected to the cannula inside the lateral ventricle; 2 – pressure transducer connected to the cannula inside the lumbar subarachnoid space; 3 – Quand Bridge; 4 – PowerLab/800; 5 – personal computer; h_c_ – distance between the cisterna magna and the pressure measuring point inside the lateral ventricle; h_s_ – distance between the cisterna magna and the pressure measuring point inside the lumbar subarachnoid space. B. Different cat's and model's body positions in which fluid pressure changes have been recorded. “c”- cranial part of the CSF system or cranium in cats; “s” – spinal part of the CSF system or spinal subarachnoid space; horizontal position −0-°; cranium or “cranial” part of the model facing upwards−45-°; cranium or “cranial” part of the model facing upwards −90-° (upright position); cranium or “cranial” part of the model facing downwards−225-°; cranium or “cranial” part of the model facing downwards −270-° (head- down position).

After setting the measuring cannulas, the cat was removed from the stereotaxic device and then fixed in a prone position on a board (Figure 1). CSF pressures were recorded using pressure transducers (Gould P23 ID, Gould Instruments, Cleveland, OH, USA) which were connected to a system that transformed analogous to digital data (Quand Bridge and PowerLab/800, ADInstruments, Castle Hill, NSW, Australia), and then entered into a computer (IBM, White Plains, NY, USA) (Figure 1).

Pressure transducers were calibrated by use of a water column; the interaural line was taken as zero pressure. Instruments for pressure measurement were fixed on the board in such a way that the membrane of each transducer was at the same hydrostatic level as the corresponding measuring cannula, so there was no need to additionally adjust the transducers during the body position changes (Figure 1). CSF changes were recorded at 15 minute intervals in horizontal −0-°, head facing upwards −45-°; head facing upwards −90-°; head facing downwards −225-°; head facing downwards −270-° position (Figure 1B).

### Experiments on a model

**Figure 2:**
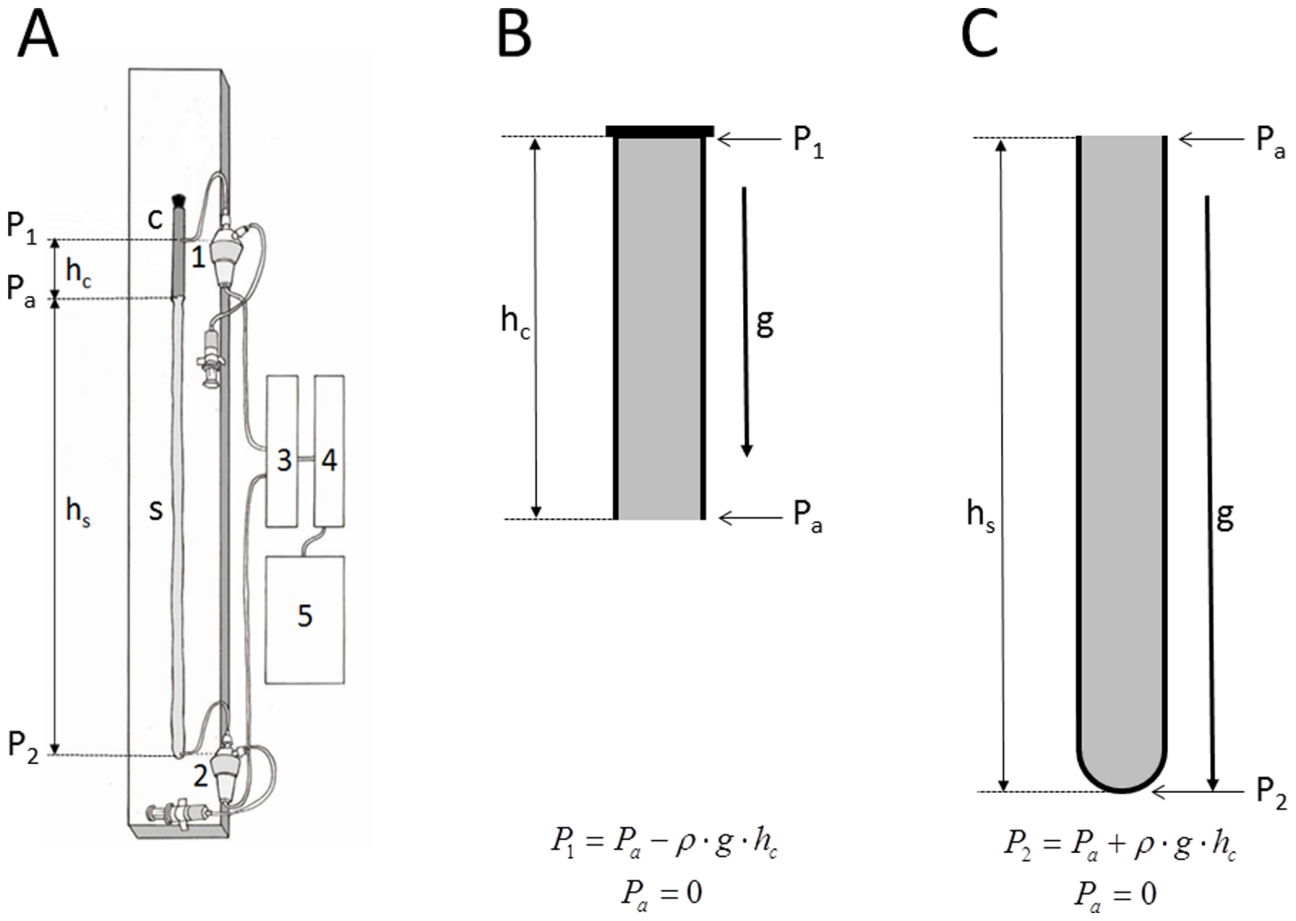
Scheme of a new CSF system model in an upright position. A. “c” represents a “cranial” part of the model (length 6 cm; gray colour) made from a plastic tube with rigid walls. “s” represents a “spinal” part of the model (length 31 cm) made from a rubber baloon. P_1_-fluid pressure in the “cranial” part of the model measured via a pressure transducer 1, which is fixed onto the board at an adequate hydrostatic level. P_a_ is atmospheric pressure, and it represents a reference pressure of 0 cm H_2_O. P_2_ is fluid pressure at the distal end of the “spinal” part of the model, recorded via a pressure transducer 2, which is fixed onto the board at the same hydrostatic level. “h_c_” - distance between open end of the “cranial” part of the model and the pressure measuring point. “h_s_” – distance between open end of the “cranial” part of the model and the pressure measuring point inside of the “spinal” part of the model. 3 – Quand Bridge; 4 – PowerLab/800; 5 – personal computer. B. Scheme of a “cranial” part of the CSF system model. g – gravitational force; ρ - is fluid density; “h_c_” - distance between open end of the “cranial” part of the model and the pressure measuring point; P_1_ – fluid pressure at the top of the plastic tube with open end on its bottom; P_a_ – atmospheric pressure. C. Scheme of a “spinal” part of the CSF system model. g – gravitational force; ρ - is fluid density; “h_s_” – distance between open end and the pressure measuring point inside of the “spinal” part of the model; P_2_ – fluid pressure at the bottom of the “spinal” part of the model; P_a_ – atmospheric pressure.

A new model of the CSF system is made of two different materials which represent the main biophysical characteristics of the cranial (unchangeable volume) and spinal (changeable volume) part of the CSF system (Figure 2). In the construction of the CSF system model, we took into account the anatomical dimensions of the CSF system in cats. The “cranial” part is made of a plastic tube, 6 cm long, with an inner diameter of 0.6 cm and wall thickness of 2.0 mm. This length of the plastic tube with a rigid wall is chosen because it represents the mean distance from the frontal sinuses to the foramen magnum, as found in 5 cats on x-rays of the animals' skulls [30]. The “spinal” part is made of a rubber balloon that is 31 cm in length (Natural Rubber Latex, Gemar, Casalvieri, Italy). This length is similar to the mean distance between the cisterna magna and the lumbar subarachnoid space at the level L_3_ vertebra where the pressure in cats was measured. The measuring cannula in the “cranial” part of the model was proximally placed 4 cm (h_c_; Figure 2A) from the lower end of the plastic tube, which corresponded to the distance between the cranial cannula in LV and the foramen magnum in cats. The second cannula was placed at the base of the rubber balloon so that the total distance between the two measuring cannulas was 35 cm (h_c_+h_s_; Figure 2A).

Before measuring the pressures, the model was filled with artificial CSF without the presence of air bubbles and fixed on a board. The pressure transducers (Gould P23 ID, Gould Instruments, Cleveland, OH, USA) were fixed at the same level as the measuring cannulas and connected to the computer via an amplifier (QUAD Bridge and PowerLab/800, ADInstruments Ltd., Castle Hill, NSW, Australia) (Figure 2A). The pressures were measured in the same positions as in the cats (horizontal −0-°; “cranial” part facing upwards −45-°; “cranial” part facing upwards −90-°; “cranial” part facing downwards −225-°; “cranial” part facing downwards −270-°).

The rubber balloon that was used for creation of the “spinal” part of the model had two modules of elasticity [22]. Those modules were of the same order of magnitude as dural elasticity modules in big experimental animals [24], [25]. It was possible to stretch that balloon, especially in the horizontal plane, the same way as it is possible for the animal dura mater. Namely, in the cranio-caudal direction, dura mater is maximally stretched, while the stretching in the horizontal direction is possible because of the arrangement of collagen fibers [24], [25], [31]. This enables the influence of atmospheric pressure on the “spinal” part of the model, as it is possible in cats across the abdominal and thoracic venous vessels which drain blood from the epidural venous plexuses. Apart from this, the displacement of fluid downward in an erect position is possible, which was earlier considered in experimental works from the beginning of the twentieth century [32]–[34], in which the elasticity of elastic elements in the spinal space (dural sac, epidural venous plexuses, epidural fibers, etc.) was tested.

### Cervical stenosis in cats

In experiments on another group of cats (n = 5), the additional laminectomy of the cervical C_2_ vertebrae (5×10 mm) and the exposition of the dura was performed. Immediately after opening, a plastic semiring (width 2× length 12 mm; thickness 1 mm) covering the dorsal and lateral parts of the dura and gently pressing on the cord was positioned. We immediately covered the opening with dental acrylate, and that way hermetically isolated the system from the atmospheric pressure influence. In our previous publication, CSF pressures were recorded over a 60 minute period after performing this kind of cervical stenosis, and normal pressure values were observed despite the fact that a communication interruption was achieved [35]. In animals with cervical stenosis, CSF pressure in the LV and LSS was recorded only in the horizontal and upright positions, the same way as was previously described.

In an additional group of animals (n = 5), laminectomy (5×10 mm) of the C_2_ and L_3_ vertebrae was performed without closure of the created opening with dental acrylate, in order to observe the changes in dural width in the cervical and lumbar region during the changes in body position, without the simultaneous monitoring of CSF pressure changes. These segmental changes in animals' dural diameter were compared to the changes in diameter of the “spinal” part of the model in a “cervical” and “lumbar” region during same changes of the model “body” position.

Data are shown as a mean value ± standard error of the mean (SEM). A statistical analysis of all of the results was performed using the Paired Student's t-test and ANOVA for repeated measures, with “condition” (cranial part, lumbar part) and position (0-°, 45-°, 90-°, 225-°, 270-°) as a within subject variable. A 2×2 mixed ANOVA was conducted on CSF pressure in a head up position (90-°), with “condition” (cranial part, lumbar part) manipulated within-subjects and model vs. animal as a between-subjects variable. P<0.05 was considered as statistically significant. All statistical analysis was performed using the SPSS 20.0.0 software (IBM Corp., Armonk, NY).

## Results

**Figure 3:**
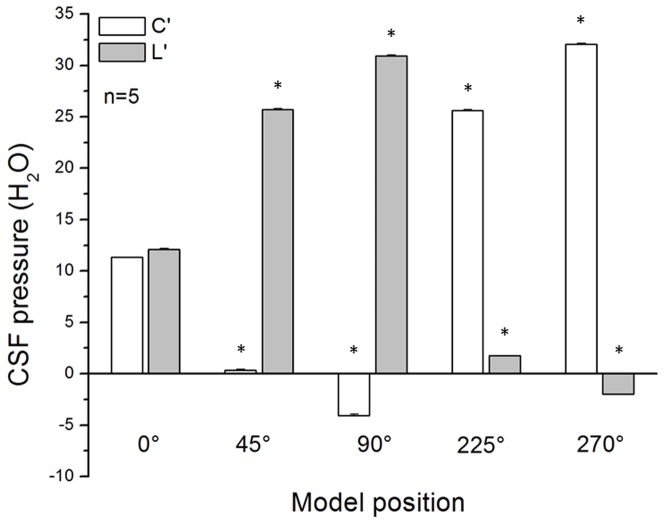
Effects of the model position changes (horizontal −0-°; “cranial” part facing upwards −45-°; “cranial” part facing upwards −90-°; “cranial” part facing downwards −225-°; “cranial” part facing downwards −270-°) on the fluid pressure (cm H_2_O) inside the “cranial” (C') and the “lumbar” (L') part of the model (n = 5). Results are shown as a mean value ± standard error of the mean (SEM; *p<0.001).

Results in Figure 3 show the changes in the mock CSF pressure measured inside the “cranial” and “lumbar” part of the model during different model positions (horizontal −0-°; “cranial” part facing upwards −45-°; “cranial” part facing upwards −90-°; “cranial” part facing downwards −225-°; “cranial” part facing downwards −270-°). The results of 5 measurements show that the pressures inside the “cranial” (11.3±0.1 cm H_2_O) and “lumbar” (12.1±0.1 cm H_2_O) parts of the model in a horizontal position are similar. When we lift the model up from the horizontal position so the “cranial” part is facing upwards, the pressure inside the “cranial” part reduces. Therefore, the mock CSF pressure inside the “cranial” part in a position of 45-° amounts to 0.3±0.1 cm H_2_O (p<0.001), while in a position of 90-° it is reduced to negative values and amounts to −4.1±0.1 cm H_2_O (p<0.001). The mock CSF pressure inside the “lumbar” part of the model in a position of the “cranial” part facing upwards−45-° increases, compared to the values in a horizontal position, and amounts to 25.7±0.1 cm H_2_O (p<0.001), while in a position of the “cranial” part facing upwards−90-° it becomes even higher and amounts to 30.9±0.1 cm H_2_O (p<0.001). During the changes it can be observed that the “cervical” part of the model is somewhat narrower than the “lumbar” part of the model. When we place the model in a reversed position, i.e., in a position with the “cranial” part of the model facing downwards−225-°, the mock CSF pressure inside the “cranial” part of the model increases compared to the one in a horizontal position, and amounts to 25.6±0.1 cm H_2_O (p<0.001), reaching even a higher value (32.1±0.1 cm H_2_O; p<0.001) in a position of the “cranial” part facing downwards−270-°. The pressure inside the “lumbar” part of the model decreases to values of 1.8±0.0 cm H_2_O (p<0.001) when we place the model in a position of the “cranial” part facing downwards−225-°, and it becomes negative (−2.0±0.0 cm H_2_O; p<0.001) when the model is in a position of the “cranial” part facing downwards −270-°. In that position, we can observe a collapse of the “lumbar” part of the model (redistribution of the fluid volume from the hydrostatically higher to the hydrostatically lower parts of the model's “spinal” part), i.e., it can be seen that the “cervical” part is wider than the “lumbar” part. Based on all of this, it can be concluded that the observed changes of fluid pressure inside the model correlate with an anatomical distance (35 cm) between the cannulas inserted into the “cranial” and the “spinal” part.

**Figure 4:**
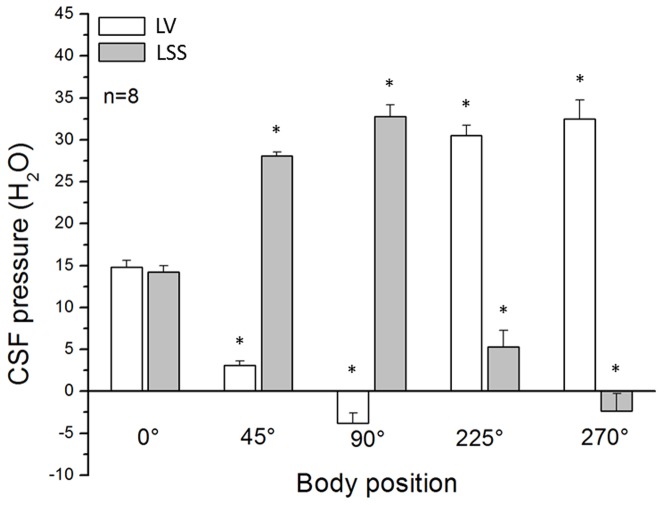
Effects of body position changes (horizontal −0-°, head facing upwards −45-°; head facing upwards−90-°; head facing downwards −225-°; head facing downwards −270-°) on CSF pressures (cm H_2_O) inside the lateral ventricle (LV), and the lumbar (LSS) subarachnoid spaces in 8 cats. Results are shown as a mean value ± SEM (*p<0.001).

We fixed an anaesthetized cat to a wooden board in different positions (horizontal −0-°, head facing upwards −45-°; head facing upwards −90-°; head facing downwards −225-°; head facing downwards −270-°), which matched the positions used with the model, and concomitantly measured the CSF pressure inside the lateral ventricle (LV) and lumbar subarachnoid space (LSS) at the L_3_ level. The results we obtained are shown in Figure 4. When an animal is in a horizontal position, pressures in LV (14.8±0.8 cm H_2_O) and LSS (14.2±0.8 cm H_2_O) are similar. By lifting the animal into a head up position, CSF pressure inside the LV decreases so that in a head facing upwards −45-° position it has the value of 3.1±0.5 cm H_2_O (p<0.001), and in a head facing upwards −90-° (upright) position it decreases to a negative value (−3.8±1.2 cm H_2_O; p<0.001). Pressure in the mentioned positions increases at the cannula placed into LSS, and it amounts to 28.1±0.5 cm H_2_O (p<0.001) in a head facing upwards −45-° position, and to 32.8±1.4 cm H_2_O (p<0.001) in a head facing upwards −90-° position. We can observe the opposite situation when we change the cat's position from horizontal to head facing downwards −225-°, because in this situation the CSF pressure inside LV increases to the value of 30.5±2.0 cm H_2_O (p<0.001), and decreases to the value of 5.3±2.0 cm H_2_O (p<0.001) in LSS. In a head facing downwards −270-° (head down) position, an increase of CSF pressure inside LV is even more pronounced, and it amounts to 32.5±2.3 cm H_2_O (p<0.001), while the measuring cannula set in LSS shows negative values of −2.4±2.1 cm H_2_O (p<0.001).

**Figure 5:**
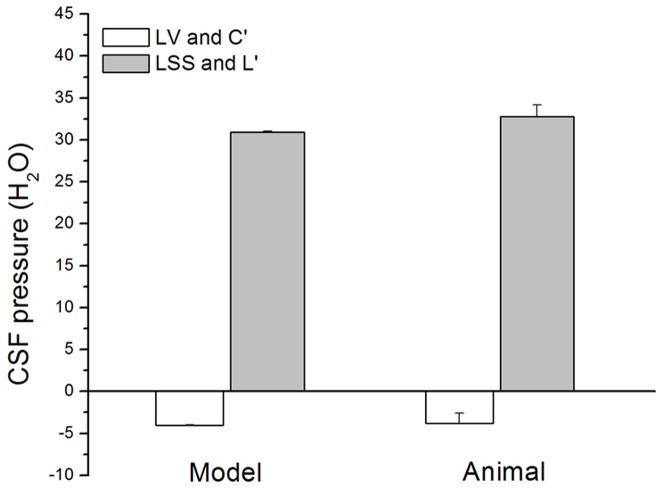
Fluid pressure inside the “cranial” (C') and the “lumbar” (L') part of the model (n = 5), and CSF pressure inside the lateral ventricle (LV) and lumbar subarachnoid space (LSS) in cats (n = 8) in an upright position. Results are shown as a mean value ± SEM (p>0.05).

In a separate series of experiments on cats, we made apertures 5×10 mm in size on the corpuses of the C_2_ and L_3_ vertebrae so we could monitor the changes in width of intact dura in these regions, depending on the changes of body position. We observed the same phenomenon as in a model. Namely, it seems that the appearance of negative pressure inside the lumbar sac in cats in a head down position is a consequence of dural sac collapsing, and redistribution of the CSF volume inside the spinal dural sac (at the same time widening of the cervical dura can be observed). Contrary to that, in a head up position, narrowing of the cervical dura and widening of lumbar dura can be observed. For the purpose of comparison, data of the pressures obtained in a model and in anaesthetized animals in the position with “cranial” part of the model facing upwards at 90-° and in a head facing upwards at 90-° position (Figures 3 and 4) are shown together in Figure 5.

It can be seen that pressure values measured in a model and in animals in mentioned positions do not differ (p>0.05). Namely, when processing the data, it was revealed that there is no statistically significant difference (p>0.05) between the pressures in the correlating parts of the model and of anaesthetized animals. Since almost the same values can be obtained from the model and from animals, those results implicate that the pressure changes in animals during changes of body position depend on the biophysical characteristics of the craniospinal system, and not on an active regulatory processes such as secretion and circulation.

**Figure 6:**
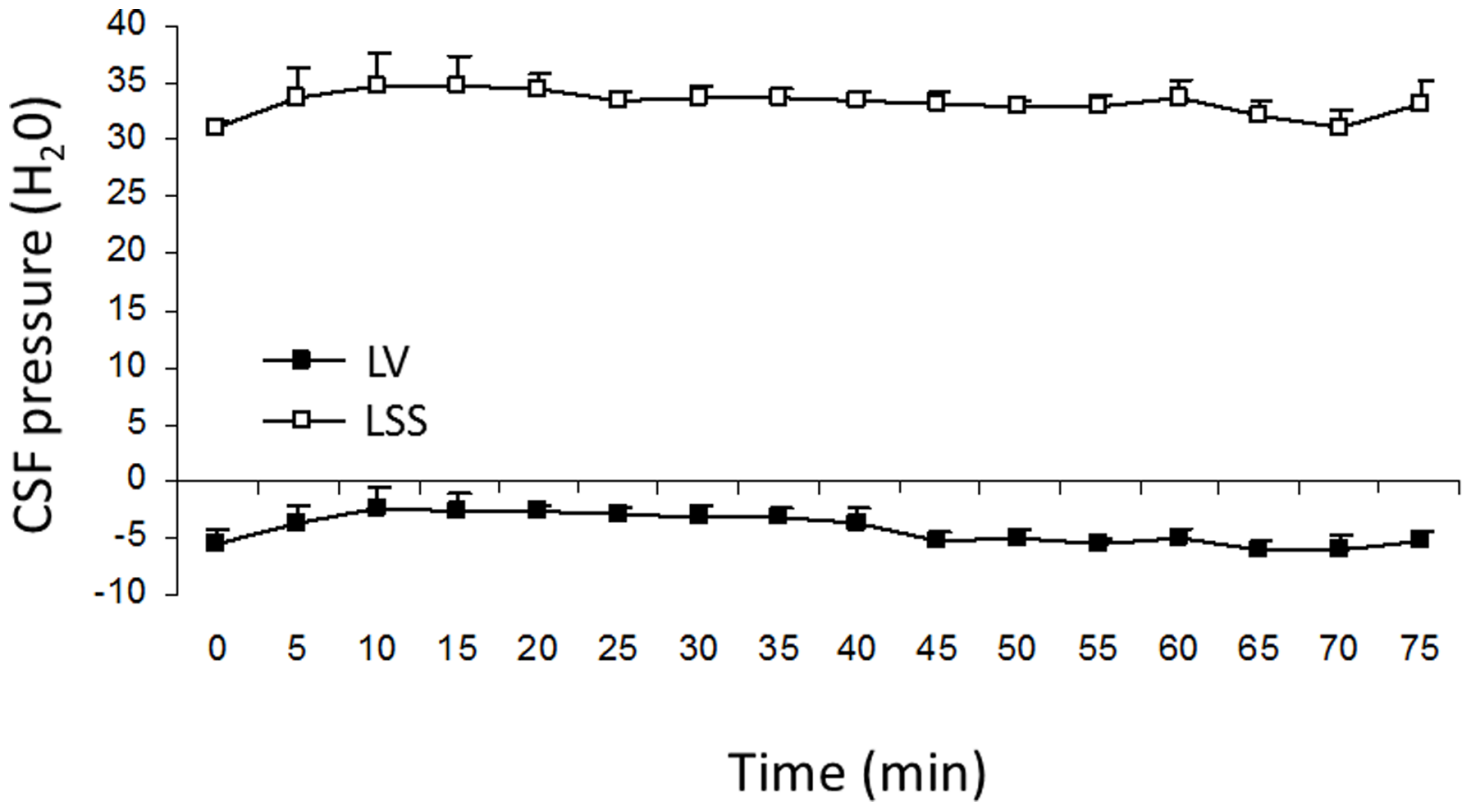
CSF pressure changes inside the lateral ventricle (LV) and the lumbar subarachnoid space (LSS) in cats in upright position. In five cats (n = 5) CSF pressure was measured during 50 minute period, and in four cats (n = 4) during 75 minute period. Results are shown as a mean value ± SEM.

Figure 6 shows results obtained from experiments in which we kept cats fixed on a measuring board (see Materials and Methods) in an upright position (head facing upwards at 90-°) for a period of 75 minutes. The CSF pressure values in LV varied between −2.8±1.6 cm H_2_O and −6.1±1.3 cm H_2_O. In LSS, the values were between 31.18±0.5 cm H_2_O and 34.8±2.6 cm H_2_O. It can be seen that during the experiment CSF pressure values inside LV were lower than the atmospheric pressure. Although the pressure values varied to some extent, it can be observed that anatomical correlation (36–37 cm) was preserved at every measuring interval. Apart from the CSF pressure, animal's arterial pressure was also monitored, and in this position it varied within the control values of 14±0.4 and 16±0.3 kPa. This indicates that in an upright position intracranial pressure is not temporarily lower than the atmospheric pressure.

**Figure 7:**
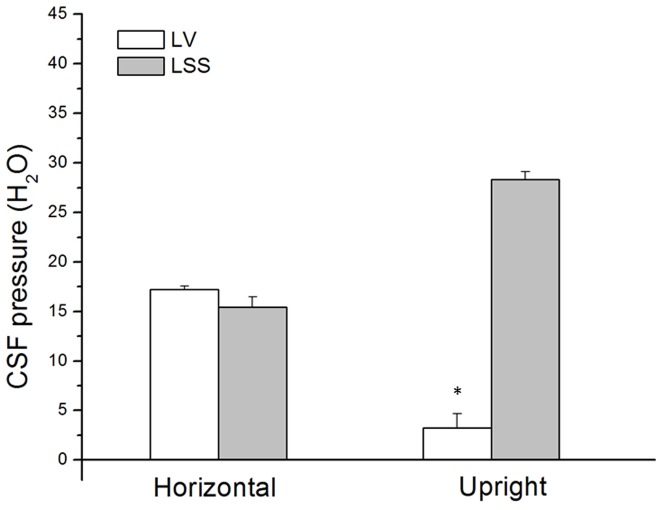
CSF pressure changes inside the lateral ventricle (LV) and the lumbar subarachnoid space (LSS) in cats with cervical stenosis (n = 5) in horizontal and head facing upwards at −90-° (head-up) position. Results are shown as a mean value ± SEM (*p<0.001).

In Figure 7, changes in the CSF pressure are shown inside LV and inside LSS in cats with cervical stenosis in horizontal and upright body positions (n = 5). In the horizontal position, pressures inside LV and LSS are similar (17.2±0.4 and 15.5±1.0 cm H_2_O), while in the position head facing upwards at 90-°, CSF pressure inside LV was +3.3±1.4 cm H_2_O (p<0.001), and inside LSS it was 28.3±0.8 cm H_2_O (p<0.001). Unlike cats that have an intact CSF pathway, in this group CSF pressure inside the cranium, i.e. in an upright position, is positive (pressure difference p<0.001), which is in accordance with the law of fluid mechanics (see Discussion and File S1).

## Discussion

### CSF pressures in the craniospinal space during changes of body position

**Figure 8:**
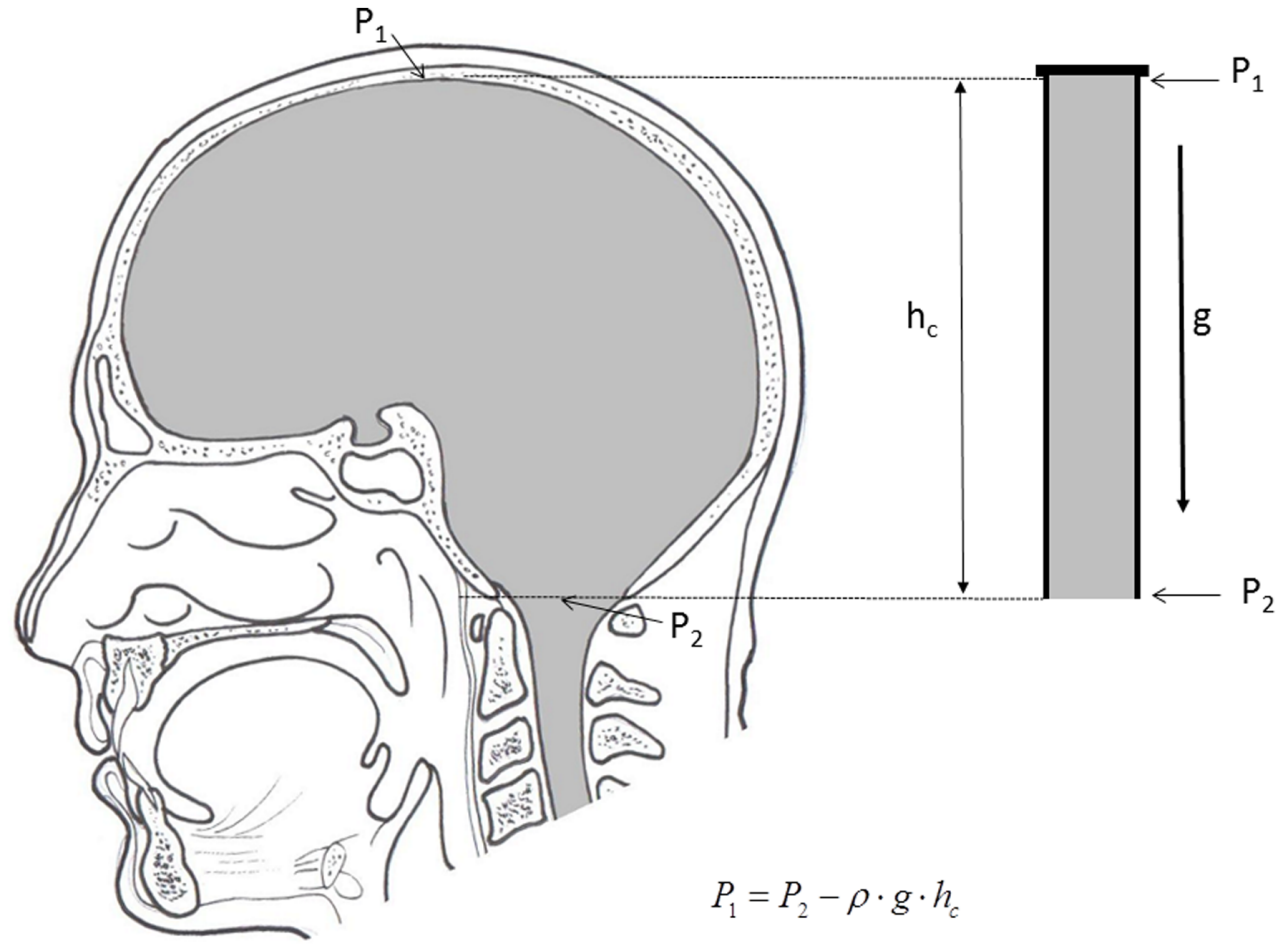
Schematic presentation of the hypothesis by which the appearance of negative CSF pressure inside the cranium in an upright position is being explained, without the changes of intracranial fluid volume. On the right side of the scheme a plastic tube is shown, filled with fluid and open at the lower end (as in the Figure S4b in File S1). Fluid pressure at the top of the tube (P_1_) is lower than the atmospheric pressure (P_2_), and it's value corresponds with the hydrostatic fluid column inside the cylinder (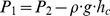, File S1). Thus, according to the law of fluid mechanics, inside that kind of space negative pressure appears without the changes of the fluid volume. According to the mentioned law, CSF inside the cranium should undergo the same fate (File S1). Namely, according to this law, negative value of the hydrostatic CSF pressure inside the cranium does not depend on the shape of the volume (File S1), but only on the distance between the point of measurement and foramen magnum (h_c_).

Fluid pressure changes obtained on our new model, which mimics the cerebrospinal fluid system in cats by its anatomical dimensions and basic biophysical characteristics of the cranial and spinal intradural space are, in fact, not different from CSF pressure changes obtained on animals during the changes of body position (Figures 3, 4 and 5). According to that fact, we hypothetized that the hydrostatic CSF column inside the cranium should follow the fate of fluid inside a rigid tube that is open at one end, which is in accordance with the law of fluid mechanics (Figure 8; File S1, Figure S4 in File S1). In other words, a firm bony armor that is open at one end (foramen magnum) imitates a rigid tube filled with water, as is shown in Figure 8. According to the mentioned law, fluid pressure (P) inside that rigid tube is lower than the atmospheric (Pa) pressure (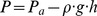; Figure 2B and 8). In addition, the value of that fluid pressure corresponds to a hydrostatic column (h) inside the rigid tube. Based on the above mentioned, we would expect that inside the cranium during body verticalization CSF pressure should be negative, and that pressure values should correspond to anatomical difference between the site of pressure measurement (for example, LV) and foramen magnum (Figure 8; File S1, Figure S4 in File S1). Further in the past, some scientists also indicated the existence of negative pressure inside the cranium by means of theoretical analysis [36]. However, the fate of fluid inside the “spinal” part of our model is different from that in the “cranial” part in an upright position, due to the influence of atmospheric pressure and the distensibility of the“spinal” part. We expect fluid pressure in that part of a model to be higher than the atmospheric pressure (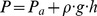; see File S1; Figure 2C), and it's value should be the same as the distance (h) between opening end of a distensible tube and the pressure measuring point.

In our new model, the pressure measuring cannula was placed 4 cm from the open end of that tube (see Materials and Methods). Thus, during verticalization of the model we would expect, based on the observation of the fluid movement inside this kind of tube, that the pressure inside the “cranial” part should be negative, i.e., that it should amount to about −4 cm H_2_O. The mock CSF pressure inside the “cranial” part of the model in a position with the “cranial” part facing upwards−90-° was −4.1±0.1 cm H_2_O (Figure 3), which corresponds to expected result. For above mentioned reasons, we expect that the pressure at the bottom of model's “spinal” part during it's verticalization would be positive, and correspond to hydrostatic difference between the site of pressure measurement and the site of connection between the “cranial” and the “spinal” part of our model. The length of the model's “spinal” part amounted to 31 cm, and the pressure measured at its bottom, in the position with the “cranial” part facing upwards −90-°, was +30.9±0.1 cm H_2_O (Figure 3), which also corresponds to the expected result.

In cats, the CSF pressure measuring cannula was placed inside the lateral ventricle at a distance of 4 cm from cisterna magna, i.e., from foramen magnum. If our hypothesis is correct, we would also expect negative pressure to appear inside the cranium during animal verticalization, and that it would amount to approximately −4 cm H_2_O, which corresponds to the obtained results (Figure 4; −3.8±1.2 cm H_2_O in a head facing upwards −90-° position). Figure 5 shows that there is no statistically significant difference between the results obtained on a model and those obtained on an animal. This fact suggests that in a vertical position negativity of the pressure inside the cranium is a consequence of primarily biophysical characteristics of the firm cranium which contains free CSF.

Similar to our model of the “craniospinal” system, in the lumbar subarachnoid space of cats during verticalization there is an increase in CSF pressure. In our group of experimental animals, an average distance between the cisterna magna and the site of pressure measurement inside the lumbar space was 32.3±0.4 cm, and pressure obtained in a head facing upwards −90-° position was 32.8±1.4 cm H_2_O (Figure 4). Thus, that pressure value corresponds to the hydrostatic height of a CSF column inside the spinal subarachnoid space, which is also in accordance with our new hypothesis.

In a number of studies [9]–[12], [37]–[41] it was noticed that when the body is vertical there is a decrease in pressure to subatmospheric values inside the cranium, together with a simultaneous pressure increase inside the lumbar area. It is generally believed that this CSF pressure decrease inside the cranium is transitory [14], [15], [20], [21], [42] because this is in accordance with the classical concept of CSF secretion, circulation and absorption [1], [2], [8], [16]. However, with humans in a sitting position, CSF pressure values inside the lumbar space never reach the value that we would expect based on the hydrostatic difference between the top of the cranium and the lumbar space. It is well known from numerous studies [9]–[15] that pressure value inside the lumbar space is approximately the same as a hydrostatic difference between cisterna magna and lumbar space, i.e., that hydrostatic CSF pressure value is zero (level of atmospheric pressure) in the cervical region. Thus, it seems, according to the observed results, that the cranial CSF column in an upright position is “disconnected” and does not contribute to the total hydrostatic fluid pressure measured with lumbar cannula, even if the measurements are made when in a sitting position over a one hour period [14], [15]. We believe (according to our hypothesis) that this “disconnection” is a consequence of the fact that cranial CSF cannot pass from cranial into spinal part of the CSF system under the influence of gravity during verticalization.

In addition, if intracranial CSF pressure during body verticalization changes in accordance with the law of fluid mechanics, than cranial CSF pressure in the case of cervical stenosis in cats should be positive in an upright position, because in that case stenosis forms a nondistensible bottom of the fluid column, which significantly changes hydrostatic and hydrodynamic relations inside the cranium (File S1, Figure S4c in File S1). Results obtained from experiments with cervical stenosis (Figure 7) indicate that during animal verticalization there is no appearance of negative intracranial pressure, on the contrary, this pressure is positive, which is in accordance with the theoretical consideration related to fluid mechanics presented in the File S1 (Figure S4c in File S1; equation S12 in File S1). Observations of ICP changes in patients with a cervical blockade that change their position from horizontal to vertical [14], [15], [42] also support our hypothesis. Namely, in those patients, a slight decrease in CSF pressure inside the cranium (without “transient” subatmospheric fall of CSF pressure), and a slight and delayed increase of CSF pressure inside the lumbar area can also be observed, compared to the patients without a blockade.

### Is subatmospheric (negative) intracranial CSF pressure normal in an upright position?

According to our hypothesis, CSF pressure depends on fluid mechanics, and negative CSF pressure should not be transitory, but it should stay negative the whole duration of the experiment. Namely, as shown in Figure 6, negative pressure value inside the lateral ventricle of cats in an upright position is always continually negative (it varies from −2.8 to −6.1 cm H_2_O) throughout the entire experiment duration (75 minutes), while at the same time, the value of CSF pressure inside the lumbar subarachnoid space is positive. It is important to emphasize that anatomical relations, i.e., hydrostatic difference between the pressures inside the cranium and lumbar space, have stayed the same (36–37 cm H_2_O) throughout the entire experiment duration (regardless of the fact that pressure slightly increases or decreases during measurement), which additionally corroborates the fact that CSF pressure changes happen in accordance with the law of fluid mechanics. Same result was also obtained on a single animal experiment in which pressures were monitored even over a 150 minute period. Thus, these results strongly suggest that the appearance of negative CSF pressure inside the ventricles is not transitory, but it lasts for as long as the cat is being held in an upright position.

Based on the subsequent data analysis, it can be concluded that CSF in humans also behaves according to the laws of fluid mechanics (our hypothesis). In the cases when CSF pressure was measured for a longer period of time (60 min) in sitting subjects [14], [15], it was found to be at atmospheric pressure level (zero CSF pressure) in upper cervical region or at the level of foramen magnum, and simultaneously, CSF pressure inside the lumbar region was positive, with a value corresponding to the distance (in cm) from cisterna magna to the measuring site in the lumbar region. Thus, constant values exist for the entire observed period (60 min), and throughout this entire period CSF pressure value in the cervical area (foramen magnum) is about 0 cm H_2_O, while inside a lumbar region it is highly positive (around +60 or +70 cm H_2_O). This raises a question: What is the value of pressure inside the cranium during this period? According to the classical concept, it has to be positive because CSF is being created (pumped) actively by choroid plexuses (secretion), and the lowest CSF pressure value should be between 5 and 7 cm H_2_O. If the pressure inside the cranium was lower than that value, CSF could not be absorbed into the venous sinuses, as the hydrostatic pressure gradient between CSF and blood would not exist [2], [8]. Since CSF system in an upright position can be simplified and imagined as a tube in which all of the CSF system compartments communicate (Figures 1 and 2B; File S1, Figure S4 in File S1) during the whole measurement period, with CSF pressure in the cervical area being zero (upper third of the tube) and 60–70 cm H_2_O in the lumbar part (bottom of the tube), at the same time CSF pressure should also be 5 to 7 cm H_2_O in the cranial part (the top of the tube). Obviously, this kind of hydrostatic pressures relations inside an open tube are unsustainable (absurd data: +7 cm H_2_O inside the cranium; 0 cm H_2_O in the cervical; and +60 cm H_2_O in the lumbar region), which suggests that CSF pressure inside the cranium cannot be positive, rather it should be negative (Figures 6 and 8; File S1) the entire time during which CSF pressure value inside the cervical area is zero.

Fluid (CSF) inside the cranium and spinal area is in an uninterrupted continuity, and subsequently, an uninterrupted continuity of hydrostatic pressure from positive to negative values should also exist. Thus, observed appearance of the measured negative CSF pressure values (which earlier were explained as transitory since they did not fit into the classical CSF physiology hypothesis) is real, and these are normal negative values that exist inside the cranium in an upright position during the entire measuring period [14], [15], [37]–[41], [44]. Also supporting the hypothesis that negative pressure inside the cranium in an upright position is not a transitory phenomenon, are experimental works on humans in which CSF pressures inside the cranium in a sitting position were measured for much longer than 60 min [39], [42]. Therefore, these zero CSF pressure values in the cervical region obtained on humans in a sitting position during measurements over a long period of time, and also the simultaneously observed negative CSF pressure values inside the cranium, are similar to our experimental results, both in animals and on an artificial model (Figures 5 and 6), and could be explained by means of the law of fluid mechanics (File S1). This means that an appearance of negative CSF pressure values does not represent a transitory change, but it represents the physiological state of CSF inside the cranium in an upright body position. This is also supported by the results of ICP telemetric measurements in 26 children during the period of 8–209 days [45], which demonstrated that negative pressure (up to −10 mmHg) appeared in an upright body position even in the absence of clinical symptoms.

### Redistribution of a CSF volume inside the spinal subarachnoid space during changes of body position

Due to high hydrostatic CSF pressure inside the lumbar region in an upright position, a widening of dural lumbar sac occurs (see Results) in cats and in the “lumbar” part of the model. At the same time, a narrowing of dural cervical space and adhesion of dura to the spinal cord in cats occurs, and the same narrowing can also be observed on a model (see Results). Studies by other authors on humans also suggest that in an upright position, due to high hydrostatic fluid pressure in the lumbar sac, dura stretches transversely, which adheres it to the vertebral canal, and simultaneously narrows the lateral epidural veins [23], [31].

This suggests that the change in body position from horizontal to upright leads to CSF volume redistribution inside the spinal dural sac from cervical parts to hydrostatically lower lumbar parts. We believe that this phenomenon is also responsible for appearance of the negative pressure inside the cranium, as CSF from the spinal part, due to this slight shift inside the cervical region, does not put pressure on CSF situated inside the cranial subarachnoid space. Regardless of the deformity of the spinal CSF column (narrowing of cervical, and widening of lumbar dura during body verticalization), according to the law of fluid mechanics, hydrostatic pressure at the bottom of the spinal dural sac will correspond to the height of the fluid (CSF) column (File S1, Figure S5 in File S1).

In their research, Alperin et al. [20], [21] monitored the direction of CSF movement inside the cervical subarachnoid space during changes of body position from horizontal to upright in humans, using an MRI device. It was observed that CSF during verticalization moves in a craniocaudal direction, followed by a narrowing of the cervical subarachnoid space, and widening of epidural veins in that region. The described phenomenon was interpreted as a consequence of CSF and blood redistribution to the hydrostatically lower spinal part, i.e., that craniospinal fluid redistribution occurs. However, our results indicate that cervico-lumbar CSF redistribution takes place, and not the cranio-spinal one. Namely, our results obtained from a model suggest that pressure changes inside the cranium and the spinal part occur without changes of a free fluid volume inside the cranium, along with volume redistribution from the “cervical” to the “lumbar” part of the model. Furthermore, we noticed that during opening of the atlanto-occipital membrane in an animal sacrificed using anesthetic in an upright position, leakage of CSF from the CM area does not occur. CSF starts leaking from cranium into the CM area only when the cannula inserted into the lateral ventricle or cortical subarachnoid space is open to atmospheric pressure. Apart from these observations, the described results of Alperin et al. [20], [21] also point to the cervico-lumbar CSF redistribution, as they, too, noticed the narrowing of cervical subarachnoid space, and not the widening that should occur if more CSF entered from the cranium into the cervical subarachnoid space due to a hypothetical craniospinal CSF volume shift. The preservation of biophysical relations of the spinal canal content, apart from narrowing of the cervical subarachnoid space, also leads to the widening of cervical epidural veins that fill this space. This is also in accordance with the observed changes in humans in an experiment done by Alperin et al. [20], [21].

In a head facing downwards −270-° position, it can be observed both on a model and on cats (see Results) that the “lumbar” part of the model and lumbar dura of the cats partly collapse, which leads to an appearance of negative pressure in that region (Figures 3 and 4). At the same time, a widening of the “cervical” segment of the model and the cervical dura in cats is also observed. This indicates that there is a lumbar-cervical volume redistribution both in a model and in cats in the head facing downwards −270-° position. Thus, the negative pressure inside the CSF system in a head facing upwards −90-° position, and also in a head facing downwards −270-° position, occurs for two completely different reasons. Negative pressure inside the cranium in a head facing upwards −90-° position is a result of previously described phenomenon (File S1), which is in accordance with our hypothesis, and occurrence of negative pressure inside the lumbar region in a head facing downwards −270-° position is a result of a slight collapse of dura onto the spinal cord tissue and nerves, due to CSF volume redistribution from lumbar to hydrostatically lower cervical region.

### CSF pressure gradients inside the CSF system in different body positions and classical concept of CSF unidirectional circulation

Regardless of whether the pressure inside the cranium is positive or negative, according to the classical concept of CSF circulation, it should be the highest inside the ventricles (namely, in the middle of the cranium), then somewhat lower inside the cisterna magna (at the bottom of the cranium), and the lowest at the brain convexity (at the top of the cranial cavity). From the biophysical point of view, inside the space filled with fluid and surrounded by rigid walls (as is the case inside the cranium), this kind of hydrostatic pressure gradient is not possible to achieve, which was supported by recently published observations [46]. Namely, it was observed that CSF pressure inside the LV and CSS is negative and does not differ if measured at the same hydrostatic level inside the cranium. From the presented results it can be seen how in an upright position CSF pressure inside LV was lower than the one measured in the CM region or in the spinal subarachnoid space. The highest value of CSF pressure was observed in the lumbar part of spinal subarachnoid space (Figure 4). These data suggest that in an upright position, CSF circulation from cranial to spinal subarachnoid space is not possible. In addition, during the head facing downwards −270-° body position, CSF pressure is the highest inside the cranium and lowest inside the lumbar subarachnoid space (Figure 4), which suggests that CSF cannot circulate from the spinal subarachnoid space and CM into the cranial CSF space. Furthermore, in a horizontal position there is no hydrostatic pressure gradient inside the CSF system. Therefore, from a biophysical point of view, experimentally obtained pressure gradients inside the CSF system do not support unidirectional CSF circulation from the ventricles to the cortical, and to the spinal subarachnoid space.

It is important to stress that steady-state hydrostatic pressure gradients observed on our model, as well as on cats and patients (Figures 3, 4 and 6; [14], [15]), are in no relation to the fluid circulation, but they are created due to the influence of gravity on a CSF column inside the craniospinal system, and existence of those constant hydrostatic gradients implies that CSF does not move unidirectionally (Figure 8; File S1). Namely, those kinds of regularly observed hydrostatic gradients are only possible if CSF does not circulate, i.e., if it has negligible inertia, according to the law of fluid mechanics (File S1, Figure S4 in File S1).

Also, based on the obtained experimental results (Figures 4 and 6), it can be seen that physiological pulsatile CSF movements due to systolic-diastolic oscillations of blood volume during blood circulation along the craniospinal system [20], [21] do not influence the stability of observed CSF pressure gradients. Those pulsatile movements, according to our hypothesis of the fate of different substances inside the CSF, lead to distribution of different molecules (for example, contrast, radiolabeled proteins or polipetides, albumins, dye etc.) in all directions [27], [47], regardless of the existing hydrostatic pressure gradients. This way, substances applied into the cortical subarachnoid space can easily be distributed toward the CM and reach LV and LSS, thus, in other words, can be distributed contrary to the classically supposed CSF unidirectional circulation [27].

### Pressure and fluid physiology inside the craniospinal space during bipedal walking

In an upright position, with the appearance of negative pressure inside the ventricles and cortical subarachnoid space, the pressure inside the brain tissue should also be negative. The results of research done by Michael et al. [45] and Tschan et al. [48], who chronically monitored changes in intraparenchimal pressure by means of a telemetric system in children, also indicate that this might be true. Namely, in an upright position, pressure inside the tissue was always below zero level. The fact that changes in the brain interstitial pressure (from positive to negative values) almost simultaneously follow the changes in CSF pressure has also been shown when experimental animals were treated with hyperosmolar mannitol [49].

Thus, we believe that the results of our research pose a new and very important physiological question as to what the nature of fluid exchange on the cerebral capillary level is under these conditions (Starling's forces). These researches also indicate that cerebral perfusion pressure (CPP) values are far greater while body is in an upright position than it was previously believed. CPP is defined as a difference between arterial and intracranial pressure. In our experiments in an upright position, during the period of pressure recording arterial pressure was within normal limits, which means that CPP was higher in that body position than it was previously supposed, due to negative CSF pressure inside the cranium. Normal values of intracranial pressure are presumed to be the values of that pressure measured while in a horizontal position, i.e., values between +10–+20 cm H_2_O. Since intracranial pressure is negative while in an upright position in humans with normal cranio-spinal communication (see Figure 6), it should be around −10 cm H_2_O inside the ventricles, which indicates that CPP should be about 20 cm H_2_O higher. Thus, it seems that in healthy humans in an upright position brain perfusion is much better, and therefore, this organ is evolutionally adjusted for verticalization and bipedal walking.

The capability of the cranium to prevent significant fluid volume oscillations inside itself explains how individual animal species, that sometimes hold their heads much higher than the rest of their body (giraffes, dinosaurs…), have, or had, normal brain perfusion. This also means that the incompressibility of the cranial osseous vault, according to our hypothesis, enables constant blood brain perfusion despite sudden changes in head position during normal activity (bipedal walking, dancing, jumping, etc.). Appearance of negative CSF pressure during verticalization should also be responsible for the indentation of fontanelles in children while they are still open.

Our results and proposed hypothesis could help in explanation as to why a clinical state of patients with cranial liquorrhea is far more favorable than those with spinal liquorrhea [43]. Namely, in the case of cranial liquorrhea, during body verticalization inside the cranium even a lower subatmospheric pressure appears, which stops liquorrhea from the cranium. Contrary to that, during body verticalization in the case of spinal liquorrhea, CSF leakage continues because CSF pressure is positive inside lower parts of the spinal subarachnoid space (Figures 4, 5 and 6).

These results fit into the newly proposed concept of CSF, as well as brain and spinal cord interstitial fluid physiology, according to which volumes of these fluids are regulated with gradients of osmotic and hydrostatic forces that exist on the brain and spinal cord capillary level [28], [47], [50], [51]. In addition, this new concept was strongly supported by following water influx into CSF and brain tissue in aquaporin-1, aquaporin-4 knockout and wild-type mice, using a newly developed water molecular MRI technique based on JJ vicinal coupling between ^17^O and adjacent protons, and water molecule proton exchange [52]. According to the mentioned new concept, physiological pulsatile CSF volume movement, due to systolic-diastolic oscillations produced by blood circulation through the craniospinal space, enables the distribution of substances inside/throughout the CSF and brain tissue, contrary to existing hydrostatic pressure gradients.

## Conclusions

- Fluid pressure changes in a new CSF system model, which consists of a “cranial” part that cannot change its volume, and a “spinal” part whose volume can be changed, do not differ significantly from the fluid pressure changes inside the cranial and the spinal subarachnoid space of cats during body position changes.
- Our results suggest that negative (subatmospheric) CSF pressure inside the cranium in an upright position is a long - lasting phenomenon in healthy subjects with normal cranio-spinal communication.
- Hydrostatic CSF pressure gradients observed in all tested body positions suggest that the classically supposed unidirectional CSF circulation from the site of hypothetical secretion inside the ventricles to the main hypothetical site of absorption (into the dural sinuses of the brain convexity) is not possible from the biophysical point of view.

## Supporting Information

File S1 **Figure S1.** Small fluid element in motion and acting forces. **Figure S2.** Balance of the body and pressure gradient forces in a fluid column at rest in a tube closed at the top and opened at the bottom. **Figure S3.** Short narrow tube opened at one end immersed in incompressible fluid. **Figure S4.** Narrow tube closed at one or both ends. a) narrow tube partly immersed in incompressible fluid; and b) totally out of the container with opening at the top; and c) closed at both ends. **Figure S5.** Hydrostatic pressure in tube with different shapes. A and B represent “spinal” part; C and D represent “cranial” part of CSF system model.
(DOCX)
