# Supplementary material for: The Influence of Body Position on Cerebrospinal Fluid Pressure Gradient and Movement in Cats with Normal and Impaired Craniospinal Communication
Source: PLoS One. 2014 Apr 18;9(4):e95229. doi: 10.1371/journal.pone.0095229 (PMC3991613; doi:10.1371/journal.pone.0095229)
Supplement: File S1 — Figure S1. Small fluid element in motion and acting forces. Figure S2. Balance of the body and pressure gradient forces in a fluid column at rest in a tube closed at the top and opened at the bottom. Figure S3. Short narrow tube opened at one end immersed in incompressible fluid. Figure S4. Narrow tube closed at one or both ends. a) narrow tube partly immersed in incompressible fluid; and b) totally out of the container with opening at the top; and c) closed at both ends. Figure S5. Hydrostatic pressure in tube with different shapes. A and B represent “spinal” part; C and D represent “cranial” part of CSF system model. (DOCX) [file pone.0095229.s001.docx]

**Supporting information**

**Klarica M et al.**

**The influence of body position on cerebrospinal fluid pressure gradient and movement in cats with normal and impaired craniospinal communication**

A physical explanation about the appearance of negative (subatmospheric) pressure in the considered model of the cerebrospinal fluid (CSF) system can be given by using basic laws of fluid mechanics. The balance force for a small fluid element moving in a tube (see Figure S1) is generally given with equation S1 [1,2].

(S1)

This is basically the Newton’s 2^nd^ law that is applied to the mass (*Δm*) of a small fluid volume (*ΔV*) which is exposed to different forces. Inertial force ($\Delta m\cdot\vec{a}$,$\vec{a}$is acceleration defined as change in velocity in time) on the left side of equation S.1 is equal to the sum of the resultant body force ($\vec{F}_{B}$) caused by the fluid element weight (gravitation) and always directed down (negative z-direction in the Figure S1), resultant pressure gradient force ($\vec{F}_{PG}$) caused by the pressure difference between two points ($\Delta P=P_{1}-P_{2}$) which acts on the outside surfaces, and resultant viscous force ($\vec{F}_{V}$) caused by friction between the small fluid elements when elements are moving (see Figure S1). Equation S1 can be applied in general cases whether or not the fluid element is moving. When the fluid is not moving, then the inertial force and viscosity force are zero.


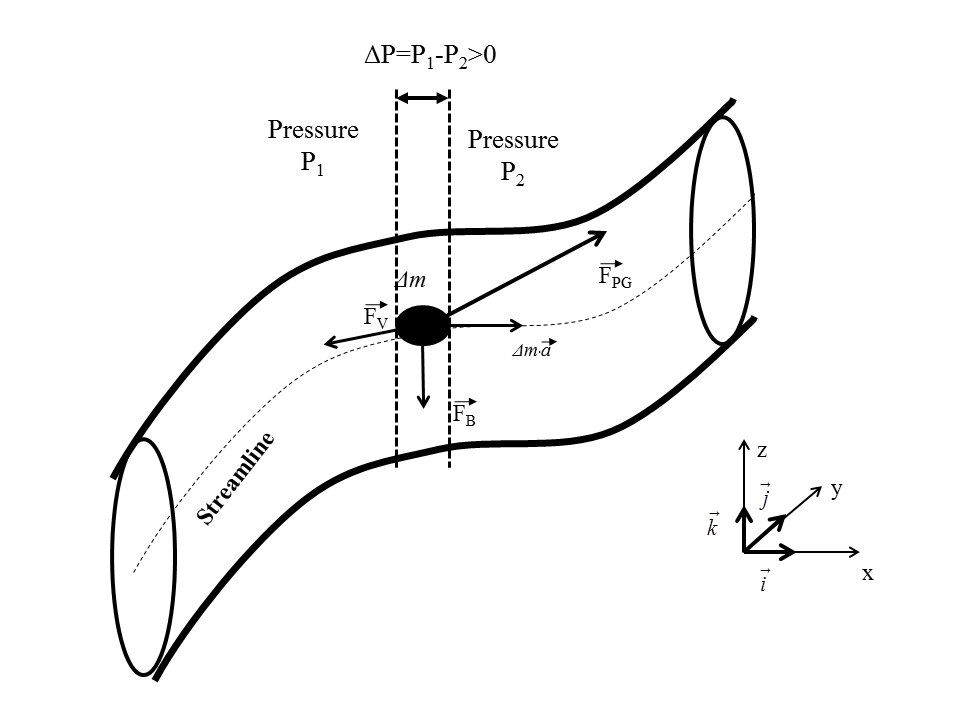


**Figure S1.** Small fluid element in motion and acting forces

Equation S1 can be divided by a small volume of fluid element (*ΔV*) to obtain an expression for forces per unit volume. Equation of motion (S1) is then given in the form of eq. S2 where ρ is fluid density ($\rho=\frac{\Delta m}{\Delta V}$).

(S2)

The body force in this consideration is caused by the effect of the gravitational field on a small fluid element ($\vec{F}_{B}=\Delta m\cdot\vec{g}$), where the magnitude of the gravitational field at the surface of the Earth is . Since the positive direction of unit vector $\vec{k}$is upward, the body force per unit volume is given with eq. S3 and it is negative in z-direction.

(S3)

The pressure gradient force per unit volume of considered fluid element can be expressed by eq. S4, using Hamilton’s (nabla,) operator applied on the scalar pressure field ().

(S4)

where are unit vectors in *x*, *y*, and *z* direction, respectively [1]. The pressure gradient force per unit volume has the opposite direction from increasing pressure (positive pressure gradient) because the fluid element in a system will always move toward the point where the pressure is lower, so the direction of the pressure gradient force is opposite to the increasing pressure field.

If the fluid element is not moving in a narrow tube closed at the top and open at the bottom, as in our experiments on the new cerebrospinal fluid model (see Figure S2), equation S2 can be simplified by including only the pressure gradient forces per unit volume and body force (gravitation) per unit volume. It is assumed that the fluid is incompressible, and in that case the density of the fluid is constant. Inertial force and viscous force per unit volume are also zero because there is no motion of fluid element.


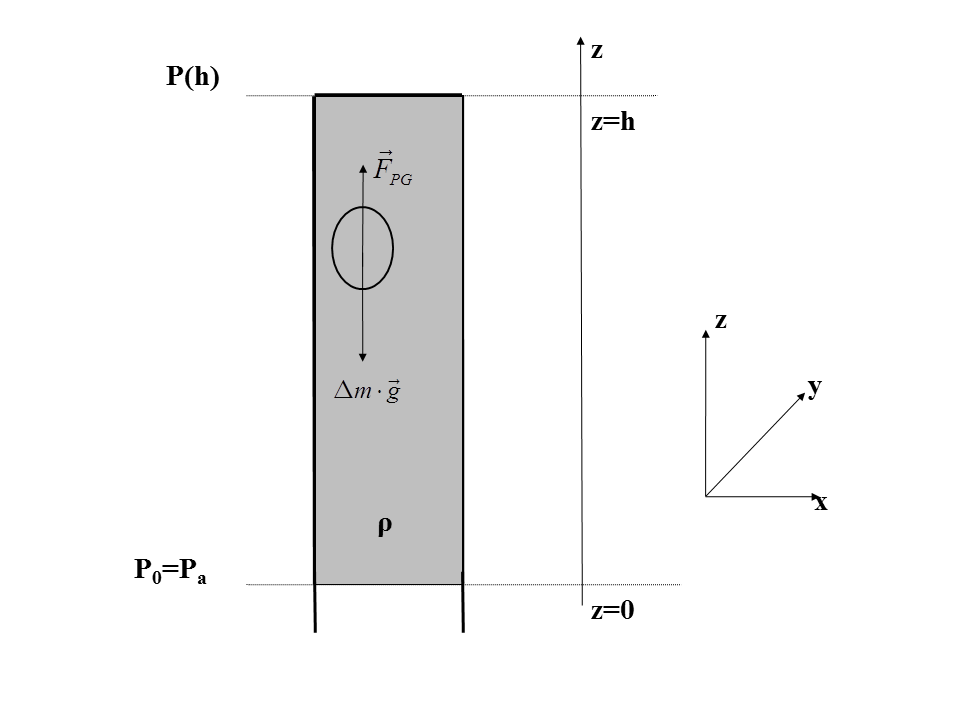
**Figure S2.** Balance of the body and pressure gradient forces in a fluid column at rest in a tube closed at the top and opened at the bottom

In that case, the force balance equation per unit volume can be written by using equation S5 [3,4] where only gravitational and pressure gradient forces are considered**.**

$0=-\rho\cdot g\cdot\vec{k}-\nabla P\to\nabla P=-\rho\cdot g\cdot\vec{k}$

(S5)

Assuming that there is no change in static pressure (*P*) in the horizontal plane ($\frac{\partial P}{\partial x}=0,\text{ }\frac{\partial P}{\partial y}=0$), the pressure differential ($\partial P$) in the fluid column in the z-direction (Figure S2) is given with eq. S6. The pressure function in z-direction can be found integrating the expression for the pressure differential

$dP=-\rho\cdot g\cdot z\to\int_{P_{0}}^{P(z)} dP=\int_{z_{0}}^{z} \rho\cdot g\cdot dz$ (S6)

where *P(z)* is pressure at position *z, P_0_* is referent pressure at position *z_0_* (in the situation shown in Figure S2, *z_0_*=0 and *P_0_=P_a_*)*.* The result of the integration is the expression for the pressure field depending on the *z* coordinate, given in the form of equation S7.

$P(z)-P_{0}=-\rho\cdot g\cdot(z-z_{0})\to P(z)=P_{0}-\rho\cdot g\cdot z$ (S7)

It can be seen that the pressure field (*P*(*z*)) at the top of the column (*z=h)* in Figure S2 is lower than the atmospheric pressure (*P_a_*) at position *z=0*. Static pressure at the top of the tube filled with incompressible fluid is then given with equation S8.

$P(h)=P_{0}-\rho\cdot g\cdot h$ (S8)

If the referent pressure in tube is chosen to be zero (in terms of gauge pressure *P_0_* =*P_a_*=0), as in our experiments, then the pressure at the top of the fluid column at rest is negative ($P(h)=-\rho\cdot g\cdot h$).

When this physical analysis is applied to our experimental models in which density and viscosity of fluid is assumed to be almost equal to the density and viscosity of water (99% of cerebrospinal fluid is water), then the appearance of subatmospheric pressure can be analyzed as follows in three different situations.

The narrow tube closed at one end and immersed in incompressible fluid in a larger container in the Earth’s gravitational field and under the influence of atmospheric pressure is shown in Figure S3.


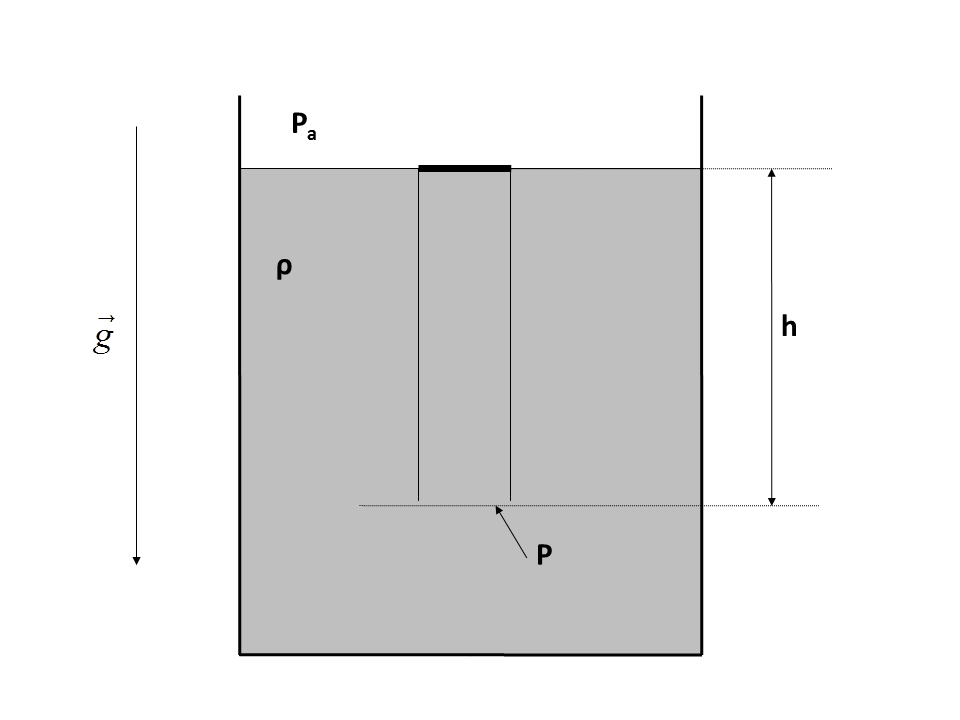


**Figure S3.** Short narrow tube opened at one end immersed in incompressible fluid

When the tube is fully immersed in incompressible fluid, the pressure at its bottom (*P* marked with arrow in Figure S3) is equal to the hydrostatic pressure in the fluid superimposed with atmospheric pressure. The hydrostatic pressure at depth *h* in the fluid is calculated as a product of density (*ρ*), gravitational field acceleration (*g*) and immersion depth (*h*), with the addition of atmospheric pressure (*P_a_*) as given with equation S9.

$P=P_{a}+\rho\cdot g\cdot h$ (S9)

Situation when the narrow tube is partly lifted above the large container with the open and closed bottom of the tube is shown in Figure S4.


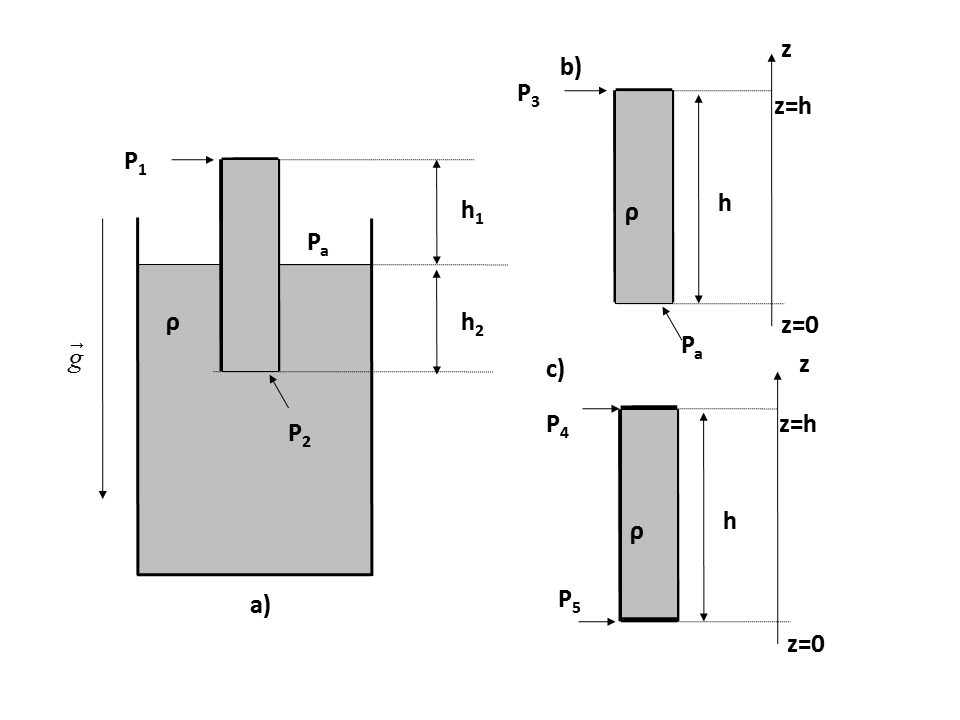


**Figure S4.** Narrow tube closed at one or both ends. a) narrow tube partly immersed in incompressible fluid; and b) totally out of the container with opening at the top; and c) closed at both ends

In the case when the narrow tube is partly lifted up (Figure S4a), the pressures at the bottom and at the top of the tube are calculated by using equations S10 and S11

$P_{1}=P_{a}-\rho\cdot g\cdot h_{1}$ (S10)

$P_{2}=P_{a}-\rho\cdot g\cdot h_{2}$ (S11)

where *h_1_* is the height of the fluid above the surface and *h_2_* is the immersion depth below the fluid surface. If the system is closed, as in our experiments, there is no influence of atmospheric pressure (*P_a_*=0).

If the tube is completely above the container (Figure S4b), then the pressure at the bottom of the tube is atmospheric pressure (*P_a_*) and the pressure at the top of the tube *P_3_* is given with equation S12.

$P_{3}=P_{a}-\rho\cdot g\cdot h$ (S12)

In the case when the narrow tube is closed at both ends (Figure S4c), the system is closed and there is no influence of atmospheric pressure at the bottom or at the top of the tube. The pressure at the top of the tube is *P_4_*=0 and the pressure at the bottom is $P_{5}=\rho\cdot g\cdot h$ because the pressure gradient force is equal to the gravitational force per unit volume.

If the tube walls are distensible, as in our experiments (spinal part of model), and the tube changes shape (see Figure S5), this does not have any influence on the considerations because the hydrostatic pressure is independent of the tube shape and volume of the above fluid [5]. The same is valid in the case when the tube is closed at the top because hydrostatic pressure in the tube does not depend on the tube shape, just on the fluid height above the measurement point (Figure S5 C and D).


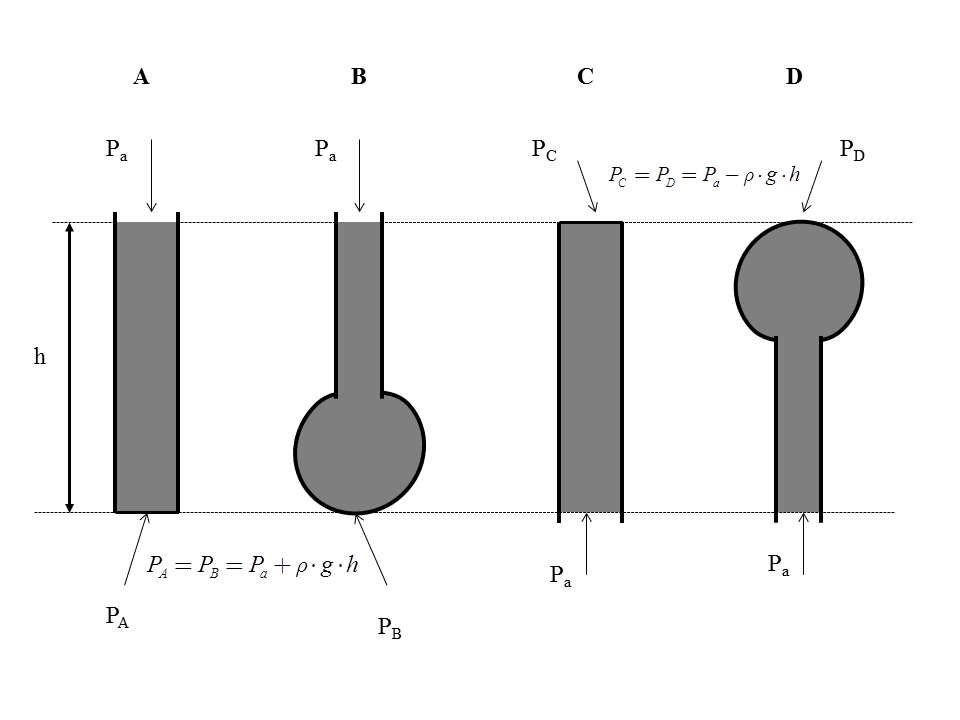
**Figure S5.** Hydrostatic pressure in tube with different shapes. A and B represent “spinal” part; C and D represent “cranial” part of CSF system model.

**References:**

1. Fox RW, Pritchard PJ, McDonald AT (2009) Introduction to Fluid Mechanics. 7^th^ ed. Hoboken: Wiley.   752 p.

2. Feynman RP, Leighton RB, Sands M (1964) The Feynman Lectures on Physics. Volume 2. Reading:  Addison-Wesley.    592 p.

3. Landau LD, Lifshitz EM (1987) Course of Theoretical Physics. Volume 6, 2^nd^  Eng. ed. Oxford:  Pergamon.  552 p.

4. Kundu PK, Cohen I M, Dowling DR (2012) Fluid Mechanics. 5^th^ ed. Waltham: Academic Press.  891 p.

5. Sears FW, Zemansky MW, Young HD (1976) University Physics. Reading: Addison-Wesley.  811 p.
